# Papaverine Enhances the Oncolytic Effects of Newcastle Disease Virus on Breast Cancer In Vitro and In Vivo

**DOI:** 10.1155/2023/3324247

**Published:** 2023-09-08

**Authors:** Sura Akram, Ahmed Majeed Al-Shammari, Hayder B. Sahib, Majid Sakhi Jabir

**Affiliations:** ^1^Department of Pharmacology, College of Medicine, Al-Nahrain University, Baghdad, Iraq; ^2^Experimental Therapy, Iraqi Center for Cancer and Medical Genetics Research, Mustansiriyah University, Baghdad, Iraq; ^3^Department of Pharmacology, College of Pharmacy, Al-Nahrain University, Baghdad, Iraq; ^4^Department of Applied Science, University of Technology, Baghdad, Iraq

## Abstract

Breast cancer is a lethal disease in females worldwide and needs effective treatment. Targeting cancer cells with selective and safe treatment seems like the best choice, as most chemotherapeutic drugs act unselectively. Papaverine showed promising antitumor activity with a high safety profile and increased blood flow through vasodilation. At the same time, it was widely noticed that virotherapy using the Newcastle disease virus proved to be safe and selective against a broad range of cancer cells. Furthermore, combination therapy is favorable, as it attacks cancer cells with multiple mechanisms and enhances virus entrance into the tumor mass, overcoming cancer cells' resistance to therapy. Therefore, we aimed at assessing the novel combination of the AMHA1 strain of Newcastle disease virus (NDV) and nonnarcotic opium alkaloid (papaverine) against breast cancer models in vitro and in vivo. *Methods.* In vitro experiments used two human breast cancer cell lines and one normal cell line and were treated with NDV, papaverine, and a combination. The study included a cell viability MTT assay, morphological analysis, and apoptosis detection. Animal experiments used the AN3 mouse mammary adenocarcinoma tumor model. Evaluation of the antitumor activity included growth inhibition measurement; the immunohistochemistry assay measured caspase protein expression. Finally, a semiquantitative microarray assay was used to screen changes in apoptotic proteins. In vitro, results showed that the combination therapy induces synergistic cytotoxicity and apoptosis against cancer cells with a negligible cytotoxic effect on normal cells. In vivo, combination treatment induced a significant antitumor effect with an obvious regression in tumor size and a remarkable and significant expression of caspase-3, caspase-8, and caspase-9 compared to monotherapies. Microarray analysis shows higher apoptosis protein levels in the combination therapy group. In conclusion, this study demonstrated the role of papaverine in enhancing the antitumor activity of NDV, suggesting a promising strategy for breast cancer therapy through nonchemotherapeutic drugs.

## 1. Introduction

Breast cancer is a heterogeneous disease with a high degree of diversity. It is the most common cancer and one of women's most important causes of death [1]. Despite the excellent antitumor activities of recent chemotherapy drugs, the treatment remains limited due to drug resistance, low therapeutic index, and severe side effects [2]. Moreover, about 40% of patients experience tumor recurrence, often as distant metastasis [3]. Therefore, combination treatments were developed to overcome cancer cell resistance and increase the antitumor effect while considering normal tissue toxicity [4]. Combination strategies involve attacking tumor cells through different mechanisms of action, which can prevent tumor cells from having the time to develop resistance to treatment and produce cell death [5]. Effective antitumor strategies require selectivity between normal and tumor cells. Therefore, choosing an oncolytic virus (OV) based on its selective replication in tumor cells with low toxicity to normal cells. Tumor cells' high susceptibility to oncolytic viruses may be due to defects in the interferon pathway or oncogenic transformation [6]. Newcastle disease virus (NDV) is a promising oncolytic virus that contains a single-stranded, negative-sense, nonsegmented RNA genome and belongs to the genus Avulavirus and family Paramyxoviridae [7]. NDV is categorized into three pathotypes: lentogenic (avirulent), mesogenic (intermediate), and velogenic (virulent), depending on the cleavage site in the F protein, which is considered a major determinant of virulence [8]. NDV-mediated oncolysis includes multiple antitumor mechanisms, such as direct cytolysis secondary to viral replication [9, 10] and apoptosis induction and activation of caspases [11–13]. Various studies revealed that NDV could induce apoptosis by activating extrinsic (death receptor-mediated apoptosis) and mitochondrial intrinsic apoptotic pathways [13]. killing mechanisms of oncolytic viruses such as immune-mediated anticancer activity, whereas. NDV infection of tumor cells leads to the expression of the viral glycoproteins (HN and F) on the tumor cell's surface, which changes the surface adhesiveness of the tumor cell to erythrocytes and lymphocytes, leading to upregulation of T-cell activation [14–16]. NDV has been shown to have potent antiangiogenic effects by triggering an acute disruption of the tumor vasculature [17] or inhibiting angiogenic factor release from cancer cells [18]. Glycolysis inhibition and cancer cell starvation were also reported to be induced by NDV [19]. Multiple challenges still face oncolytic viruses to succeed; one important point is to develop an efficient route of delivery of OVs to tumor location due to many obstacles such as normal stromal tissue of fibroblast and endothelial cells [20]. NDV is a potent apoptosis inducer in cancer cells, among other mechanisms of death induction, we selected another apoptosis inducer in cancer cells that was shown to be safe as well against normal cells to amplify the death induction by NDV in cancer cells. This is where we selected papaverine as a combined therapy to be coadministered with oncolytic NDV.

Papaverine is an ergot alkaloid isolated from *Papaver somniferum*. It is a benzylisoquinoline nonnarcotic opium alkaloid produced synthetically after isolation [21]. Unlike other alkaloids, papaverine has no analgesic or narcotic effects due to its lack of the phenanthrene group in its structure [22]. Papaverine can inhibit phosphodiesterase 10A, which increases cyclic adenosine monophosphate (CAMP) and inhibits calcium channels; it relaxes the smooth musculature of the larger blood vessels, particularly the coronary, systemic peripheral, and pulmonary arteries, and increases cerebral blood flow. The current study's rationale for combining papaverine with virotherapy is to increase the ability of the virus to reach the tumor tissue and enhance apoptosis induction, and the current study sought to investigate this hypothesis.

## 2. Materials and Methods

### 2.1. NDV Propagation

AMHA1 Iraqi strain of Newcastle disease virus stock, stored as frozen seed at −20°C was kindly provided by the Experimental Therapy Department, Iraqi Centre of Cancer and Medical Genetics Research (ICCMGR), Mustansiriyah university, Baghdad, Iraq. A stock was propagated in embryonated chicken eggs (Al-Khalil hatchery, Iraq), harvested from allantoic fluid, and then purified from debris by cold centrifugation (3000 rpm, 30 min at 4°C). NDV titers were determined by a 50% tissue culture infective dose on the vero cell line [23].

### 2.2. Papaverine

Papaverine hydrochloride was obtained from (Santacruz Biotechnology, USA)). Papaverine was stored as a 30 mM stock solution in ultrapure water at−20°C.

### 2.3. Cell Culture

The AMJ13 cell line, derived from Iraqi patient with breast cancer cells, exhibits a lack of estrogen and progesterone receptors [24]. This cell line has been widely utilized in our laboratory as well as by other researchers [25–28]. Human breast cancer cell line estrogen, progesterone receptors positive (MCF-7), were provided by the Experimental Therapy Department, ICCMGR, Baghdad, Iraq. The normal human mesenchymal cell line (hATSMCs) was kindly provided by Ahmed Majeed Al-Shammari; these cells were established from primary cultures of human samples and regularly passaged in vitro [29]. Both MCF7 and normal hATSMCs cell lines were cultured in MEM medium (US Biological, USA). In contrast, AMJ13 cells were cultured in RPMI-1640 medium (US Biological, USA); both media were supplemented with 10% (v/v) fetal bovine serum (FBS) (Capricorn-Scientific, Germany) and 1% (v/v) penicillin-streptomycin for a final concentration of 100 *μ*g/ml (Capricorn-Scientific, Germany) and incubated in a humidified atmosphere of 5% CO_2_ at 37°C.

### 2.4. Cytotoxicity Assay

AMJ13, MCF-7, and normal cells were seeded in a 96-well microplate at a density of 1 × 10^4^ cells/well and incubated overnight for 24 h at 37°C until we get a confluent monolayer, as observed under an inverted microscope. Cytotoxicity was determined by the 3-(4, 5-dimethylthiazol-2-yl)-2, 5-diphenyltetrazolium bromide (MTT) assay. The cells were exposed to a range of diluted concentrations of papaverine (10, 25, 50, 100, 150, and 200 *μ*m) (Santacruz Biotechnology, USA) and NDV over a range of diluted multiplicities of infection (0.1, 1, 3, 5, 10, and 20 MOI) for the determination of IC50 for both papaverine and NDV. After 72 h, the media was discarded, and each well received 50 *μ*l of MTT dye solution (2 mg/ml) (Bio-World, USA) was incubated for three hours and solubilized with 100 *μ*l of dimethyl sulfoxide (DMSO) (Santa Cruz Biotechnology, USA). The plates were incubated for 15 min. The optical density values of treated and untreated cells were measured at 492 nm with an ELISA plate reader [30].

(1) $$\begin{array}{c} \text{Cytotoxicity }\%=\frac{\left(\text{OD control}-\text{OD treated}\right)}{\text{OD control }x\;100}\text{.} \end{array}$$

The (OD control) is the mean of optical density for untreated cells in 3 plates, while (OD treated) is the mean of optical density for treated cells in 3 plates [31].

### 2.5. Morphological Analysis

The cell suspension was seeded in 96-well microplates at 1 × 10^4^ cells/ml density and incubated for 24 h at 37°C until the confluent monolayer was reached. To visualize cell morphology under an inverted microscope, the medium was removed, and NDV and papaverine were added. After 72 hr of exposure, the media was discarded, and 50 *μ*l of crystal violet was added to each well and incubated at 37°C for 15 min. Later, the stain was removed and gently washed with tap water three times; the plates were tilted to prevent the stream of water from hitting the cells directly, followed by cell observation under an inverted microscope at 40x magnification photographed by using a digital camera [32].

### 2.6. Combined Cytotoxicity Assays and Chou–Talalay Analysis

The doses in this experiment were selected based on IC50 obtained from the previous cytotoxicity MTT assay. We took the concentration around the IC50 value for both NDV and papaverine. The AMJ13, MCF7, and normal cell lines were seeded at a density of 1 × 10^4^ cells/well into 96-well plates and incubated overnight. NDV was added first at MOI (0.1, 1, and 2) and incubated for two hours, followed by papaverine addition at the selected concentration in micromolar (*μ*m) (10, 25, and 50 *μ*m) for the growth inhibition measurement in which each MOI of virus mixed with these three papaverine concentrations to have finally nine combinations groups. As described earlier, growth inhibition was measured after 72 h of incubation through the MTT assay. The assay was performed in triplicate. NDV and papaverine were studied at nonconstant ratios to determine synergism using the Chou–Talalay combination index (CI) calculated using the CompuSyn software (CompuSyn Inc., Paramus, NJ, USA) to analyze the combined effect of NDV and papaverine. CI values of 0.9–1, <0.9, and >1.1 indicate additive, synergism, and antagonism, respectively [33].

### 2.7. Quantification of Apoptosis Using Propidium Iodide and Acridine Orange Double Staining

Propidium iodide/acridine orange (PI/AO) dual staining was used to assess the apoptotic rates in treated and nontreated breast cancer cells and the normal cell line [34]. The cells were seeded at a density of 10,000 cells/well in an 8-well plate for 24 h before treatment and then treated with NDV at (2 MOI) and papaverine at 100 *μ*m alone and then in combination with the addition of NDV at first, and after two hours we added papaverine in a 37°C incubator for 72 h before the PI/AO staining. After incubation, the cells were washed three times with PBS and stained with 50 *μ*l of stain mixture (10 *μ*l AO + 10 *μ*l PI + 1 ml PBS) for 30 s. Finally, the dye was discarded and visualized with a fluorescent microscope [35].

### 2.8. Animal Tumor Model

The murine mammary adenocarcinoma tumor (AN3) transplantable tumor line was used in this study. It is derived from a spontaneously arising mammary tumor in an Albino Swiss mouse [36]. The AN3 tumor line is maintained by continuous transplantation in inbred syngeneic female mice. Female Swiss Albino mice which were 8–10 weeks old and 15–25 g in weight were purchased and maintained according to the guidelines of the animal house of the ICCMGR. Animal ethics approval was provided by the scientific committee of the College of Medicine, Al-Nahrain University, Baghdad, Iraq, number 5/1/52/1884 on 21/11/2019. Tumors were established by inoculating AN3 cells (10^6^/100 *μ*l per site) into the right flanks of healthy Albino mice. When the tumor nodules reached 0.5–1 cm in diameter, the experiment was started.

### 2.9. Experimental Design

The animals were randomly divided into four groups of ten: Papaverine group (injected intraperitoneally (IP) 40 mg/kg twice daily for four days [37] and Newcastle virus group injected IP, 10 × 10^6^ about 500 *μ*l every 30 minutes. The combination group was treated with both (papaverine at first, and then NDV), and the mice in the control group were left untreated until the end of the experiment. After 30 days, the mice were sacrificed by dyethyl-ether followed by cervical dislocation, and tumor tissue samples of the treated and control groups were carefully dissected and fixed in 10% neutralized buffered formalin, paraffin-embedded, and sectioned at 5 *μ*m thickness for histology and immunohistochemistry assays.

### 2.10. Evaluation of Antitumor Efficacy

The tumor diameters (length and width) were measured three times per week via caliper [38]. The tumor volume was calculated using the following formula: 0.5 × length × width × width as the mean ± SEM for each group. Mice were sacrificed when tumor burden reached a volume of 10% of their body weight. The tumor volume was normalized to the tumor volume on day zero, which was the time when the treatment was initiated.

The following equation calculated the tumor growth inhibition (TGI):

(2) $$\begin{array}{c} \text{GI}\%=\frac{\text{Tumor volume of the untreated group}-\text{tumor volume of the treated group}}{\text{Tumor volume of untreated group}}\times 100\text{.} \end{array}$$

### 2.11. Apoptosis Proteins Measurement

An immunohistochemistry assay was performed to detect apoptotic proteins in tumor tissues using the following primary mAbs: mouse anticaspase 3, 8, and 9 with a concentration of 200 mg/ml diluted at (1 : 50) (USBiological, USA), and secondary antibody: mouse antihuman IgG (Biotin) (concentration 2 mg/ml) (USBiological, USA). Immunohistochemistry was performed according to USBiological recommended procedure. The GraphPad Prism software (GraphPad, San Diego, California, USA) analyzed the results.

### 2.12. Quantitative Image Analysis

The digital pictures of immunohistochemistry were used for quantitative analysis of the hematoxylin-DAB staining slides taken with an inverted microscope and camera (Leica Microsystems, Germany); three different staining zones of ICC images of each slide were analyzed in this study. First, a color deconvolution assay was used to unmix the DAB and hematoxylin-stained areas, leaving a free image as we got three new images. The first image is the hematoxylin stain, the second is the DAB image, and we quantify the DAB image. The numerate of pixels in a specific intensity measure vs. their respective intensity was elevated utilizing the “Fiji” version of ImageJ [39]. Optical density (OD) was calculated using the following formula: OD = log (max intensity/mean intensity), where max intensity = 255 for 8 bit images.

### 2.13. Apoptosis-Related Protein Measurement for NDV-Papaverine Treatment

The apoptosis-related protein's expression response to NDV, papaverine, and combinations was measured using the RayBio® C-Series Apoptosis Antibody Array 1 kit (RayBiotech Life, Inc., USA), which detects 38 apoptotic factors. AMJ-13 cells were seeded in four tissue culture flasks (10^6^ cells per flask) and incubated at 37°C with 5% CO2. After 24 hr, the medium was removed. Three tissue culture flasks were exposed to NDV (2 moi), papaverine (100 *μ*m), and their combination for 24 hr; the untreated flask was considered a control treated with serum-free media only. Then, we added 1 ml of lysis buffer for each flask, pulled it into Eppendorf tubes, and left it for 5 min for protein extraction. Finally, the extracted proteins were calculated by nanodrop (Thermofisher, USA) and normalized. All the reagents and samples were prepared immediately prior to use.

The biomarker detection is achieved with the addition of HRP-Streptavidin; positive and negative controls are spotted within each array for assay validation. A cocktail of biotinylated detection antibodies for the apoptotic proteins was provided. Apoptotic spotted slide plastic was allowed to react with the NDV, papaverine, and combination-induced proteins with biotinylated antibodies and then reacted with HRP-Streptavidin after each addition, followed by washing with wash buffers I and II according to manufacturing recommendations. Later, 500 *μ*l of the detection buffer mixture was pipetted onto each membrane and incubated for 2 minutes at room temperature. Then, a plastic sheet was placed on top of the membrane by starting at one end and gently rolling the flexible plastic sheet across the surface to the apoptosis end to smooth out any air bubbles. The membranes were transferred to Raybiotech Life Labs, USA, for chemiluminescence imaging, data extraction, and analysis.

### 2.14. Statistical Analysis

The in vitro and in vivo results were statistically analyzed using the one-way variance test (ANOVA) analysis in GraphPad Prism (GraphPad Software, Inc. San Diego, California). The standard deviation of the mean was considered significant at *P* = 0.05. Unpaired *t*-tests were used to compare groups, and *P* < 0.05 was considered significant. A one-way ANOVA with Tukey's multiple comparisons test was performed to find the significance of AO/PI. The data in graphs are shown as the mean ± S.D.

## 3. Results

### 3.1. In Vitro Cytotoxicity of NDV and Papaverine against Breast Cancer and Normal Cells

**Figure 1:**
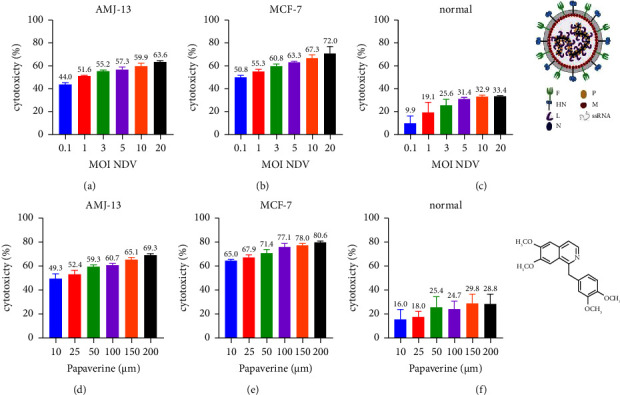
Oncolytic AMHA1 NDV and papaverine are cytotoxic against human AMJ13 and MCF-7 breast cancer cells but not cytotoxic to normal cells. The cells were treated with (a–c) NDV (MOI 0.1, 1, 3, 5, 10, and 20) and with (d–f) papaverine (10, 25, 50, 100, 150, and 200 *μ*m/ml) for 72 h. Cytotoxicity was investigated using the MTT assay and showed that IC50 values of NDV were (2 MOI AMJ-13, 1.14 MOI MCF7, and 183 MOI normal cell line) and IC50 of papaverine were (62.12 *μ*m AMJ-13, 72.62 *μ*m MCF-7, and 393.8 *μ*m on normal cell line). All data shown are the mean ± SEM from three independent experiments.

A MTT cytotoxicity assay was used to evaluate the effect of different MOIs of NDV and over a range of concentrations of papaverine on breast cancer and normal cells (Figure 1). It was found that increasing MOI of NDV and papaverine concentrations was associated with an increased cytotoxic percentage (CT%) in the breast cancer cells, but not statistically significant percentages of CT% in the normal cells, which was ranging from 44% at (MOI: 0.1) to 63.6% at MOI: 20 for AMJ-13, from 50.8% at MOI: 0.1 to 72% at MOI: 20 for MCF7, and from 9.9% to 33.4% on normal cell line (Figures 1(a)–1(c)). For papaverine, CT% ranged from 49.3% at 10 *μ*m to 69.3% at 200 *μ*m for AMJ-13 and from 65% at 10 *μ*m until it reached 80.6% at 200 *μ*m for MCF7 and from 16% at 10 *μ*m to 28.8% at 200 *μ*m for normal cell line (Figures 1(d)–1(f)). The cytotoxicity assay analysis showed that the IC50 values of NDV were (2 MOI AMJ-13, 1.14 MOI MCF7, and 183 MOI normal cell line) and the IC50 of Papaverine was (62.12 *μ*m AMJ-13, 72.62 *μ*m MCF-7, and 393.8 *μ*m on normal cell line). Therefore, we chose IC50-related doses of NDV and Papaverine for the combination study: (0.1, 1, 2 MOI) for NDV and (10, 25, and 50 *μ*m/ml) for Papaverine.

### 3.2. Combination Cytotoxicity Assays and Chou–Talalay Analysis

**Figure 2:**
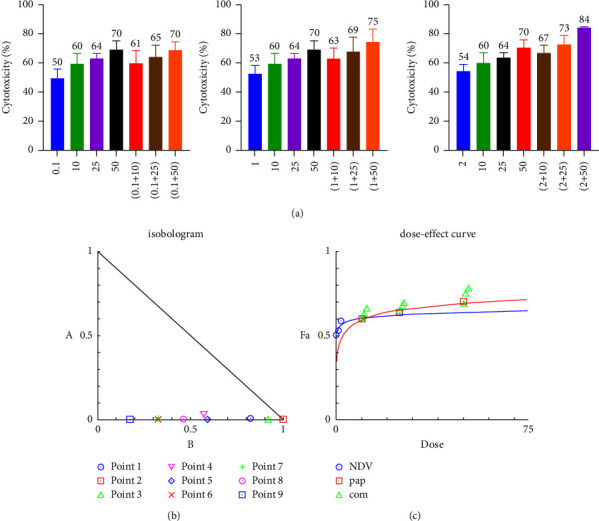
A combination of NDV and papaverine showed a superior cytotoxic effect in comparison to monotherapies in the human breast cancer AMJ13 cell line, which was treated with NDV (0.1, 1, and 2 MOI) and with (10, 25, and 50 *μ*m/ml) papaverine. Cell viability was measured by the MTT assay (a). (b-c) Illustrations of the dose-effect curve and normalized isobologram of nonconstant combination ratios were measured by the Chou–Talalay method, where the CI value quantitatively defines synergism. CI < 0.9, additive effect (CI = 0.9–1.1), and antagonism (CI > 1.1). All data shown are the mean ± SEM.

**Figure 3:**
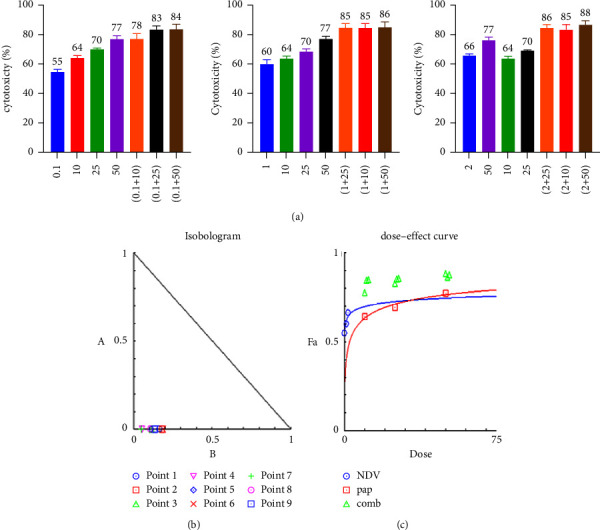
A combination of NDV and papaverine showed a superior cytotoxic effect in comparison to monotherapies in the human breast cancer MCF-7 cell line, which was treated with NDV (0.1, 1, and 2 MOI) and (10, 25 and 50 *μ*m/ml) papaverine; then, the cell viability was measured by the MTT assay (a). (b-c) Illustrations of the dose–effect curve and normalized isobologram of nonconstant combination ratios were measured by the Chou–Talalay method, where the CI value quantitatively defines synergism. CI < 0.9), additive effect (CI = 0.9–1.1), and antagonism (CI > 1.1). All data shown are the mean ± SEM.

**Figure 4:**
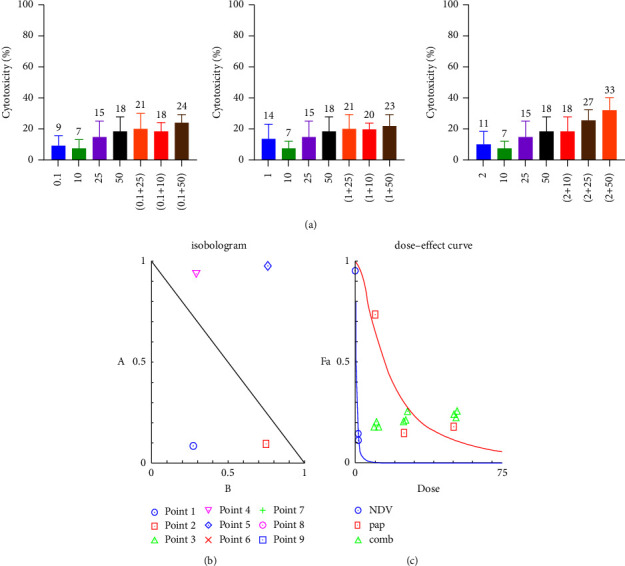
A combination of NDV and papaverine showed an insignificant cytotoxic effect as the monotherapies in the normal cell line which was treated with NDV (0.1, 1, and 2 MOI) and (10, 25, and 50 *μ*m/ml) papaverine, and then the cell viability was measured by the MTT assay (a). (b-c) Illustrations of the dose–effect curve and normalized isobologram of nonconstant combination ratios were measured by the Chou–Talalay method, where the CI value quantitatively defines synergism. (CI < 0.9), additive effect (CI = 0.9–1.1), and antagonism (CI > 1.1). All data shown are the mean ± SEM.

To evaluate the therapeutic efficacy of NDV and papaverine as a combination and its potential cytotoxicity, we examined the cytotoxicity ratio of the NDV (0.1, 1, and 2 MOI) and for papaverine (10, 25, and 50 *μ*m/ml) alone and then each MOI of NDV with three papaverine concentrations to get nine combinations. MTT cell viability assays were conducted in human breast cancer cell lines (AMJ13 and MCF-7) and normal mesenchymal cells. The combination cytotoxic effect increase was clear; for AMJ-13, the cytotoxic effect ranged from 61% for (0.1 MOI + 10 *μ*m) to 83.6% for 2 MOI + 50 *μ*m combined dose, as shown in Figure 2. A significant higher cytotoxicities were seen against the MCF-7 cell line which ranged from 77.9% to 87.8% from a combination of 0.1 moi + 10 *μ*m to 2 moi + 50 *μ*m doses, in which there was a direct relationship between the cytotoxic effect of the combination with increasing MOI of NDV and papaverine concentration (Figure 3). There were negligible cytotoxic effects on normal cells even when we increased NDV MOI dose and papaverine concentration, reaching a maximum of 32.8% at 2 MOI + 50 *μ*m papaverine (Figure 4).

The CI was estimated from the dose-effect data of single and combined treatments using the CompuSyn Isobologram. CI < 1 indicates synergism; CI = 1 to 1.1 indicates an additive effect, and CI > 1.1 indicates antagonism. The CI on AMJ-13 was synergistic at eight combination points (CI < 1) with only one additive effect. In all nine combination points, the CI against MCF-7 cancer cells was less than 1, so synergistic effects were recorded in all combination doses. Finally, on the normal cells, the combination points were higher than one in seven combinations, indicating the antagonist effect, which is a negligible effect as there were no killing effects, reaching 50% at all tested combinations (Tables 1–3).

### 3.3. Apoptosis and Morphological Changes of Breast Cancer and Normal Cell Lines

**Figure 5:**
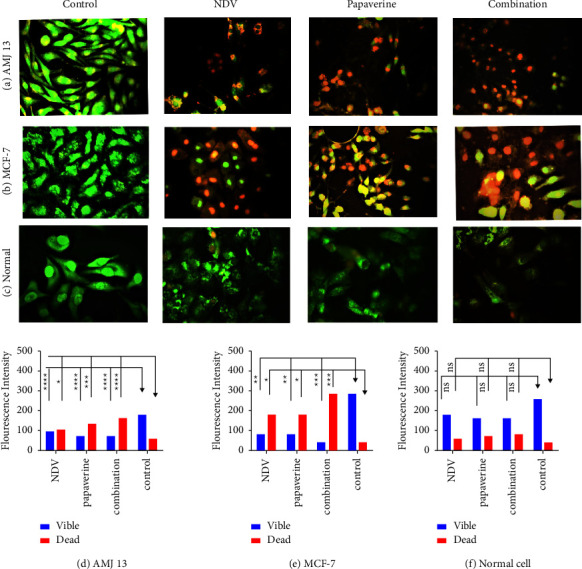
Investigation of the NDV-papaverine combined therapy to induce apoptosis in treated cells using acridine orange and propidium iodide. (a) AMJ13 and (b) MCF-7 cancer cells indicated that NDV, papaverine alone and their combination induce apoptosis, as evidenced by red-stained cells, (c) untreated control cells emitting green fluorescence cells, and (d and e) show the number of apoptotic cells is higher in the combination treatment than monotherapies (f) with insignificant differences in the number of both apoptotic and viable cells in the normal cell line. Values represent the mean ± SD. *P* ns, ^*∗*^*P* < 0.05, ^*∗∗*^*P* < 0.01, ^*∗∗∗*^*P* < 0.001, and ^*∗∗∗∗*^*P* < 0.0001. The magnification of all images was 400x.

The ability of the papaverine-NDV combination to induce apoptosis in treated breast cancer cells was confirmed via an acridine orange/propidium iodide double stain. As shown in Figure 5, apoptotic cells have been detected with an orange-red color in both breast cancer cell lines (AMJ-13 and MCF-7), which were treated with NDV and papaverine alone and in combination, while viable green cells have been detected in the control untreated and normal cell line which were emitting green fluorescence. However, NDV-papaverine-treated cancer cells were significantly emitting more red fluorescence than monotherapies (Figures 5(a)–5(c)). These results were confirmed by quantitative analysis that showed a significant increase in apoptotic cells in papaverine-NDV combination-treated cancer cells compared to monotherapies (Figures 5(d) and 5(e)), with insignificant differences in the number of both apoptotic and viable cells in the normal cell line Figure 5(f).

### 3.4. In Vivo Antitumor Effects of Papaverine-NDV Combination Therapy

**Figure 6:**
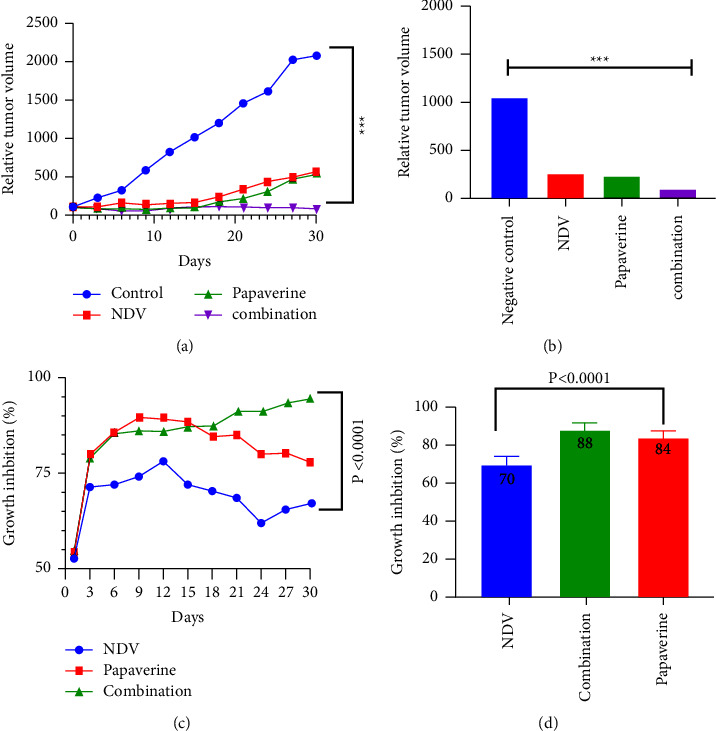
In vivo tumor growth rate in Albino Swiss mice. When the tumor nodules reached 0.5–1 cm in diameter, the animals were randomly divided into four groups of ten. (a-b) Relative tumor volumes. In response to mono and combination therapy. (c-d) Growth inhibition rates are shown for each point measured during the study period. The reduction in the combination group is greater than that in NDV and papaverine alone.

Papaverine–NDV combination antitumor activity in vivo was evaluated in an animal tumor model. The cancer cells (AN3) were injected under the skin of Swiss Albino mice to allow for tumor measurement. When the tumor mass reached 0.5–1 cm in diameter, the mice were randomly divided into four groups of ten, as described in the methods section. The relative tumor volumes were plotted over a 30-day period with day one before the treatment considered 100%. There was a continuing increase in the relative tumor size for the negative control group (without treatment) from the beginning until the end of the experiment; in contrast to the combination-treated group, the experiment ended with a clear decrease in the tumor size in which there was a significant difference between the control untreated and treated group with *p* value = 0.0008^*∗∗∗*^ (Figures 6(a) and 6(b)). Furthermore, the combination therapy group induced the highest rate of tumor growth inhibition (93.7%) at the end of the experiment, followed by the papaverine group (77.9%%), and the NDV group had the lowest rate of growth inhibition (67.50% %) with significant differences in growth inhibition between combination and mono and combination therapy with a *p* value <0.0001, as shown in Figures 6(c) and 6(d).

### 3.5. Histopathological Study

**Figure 7:**
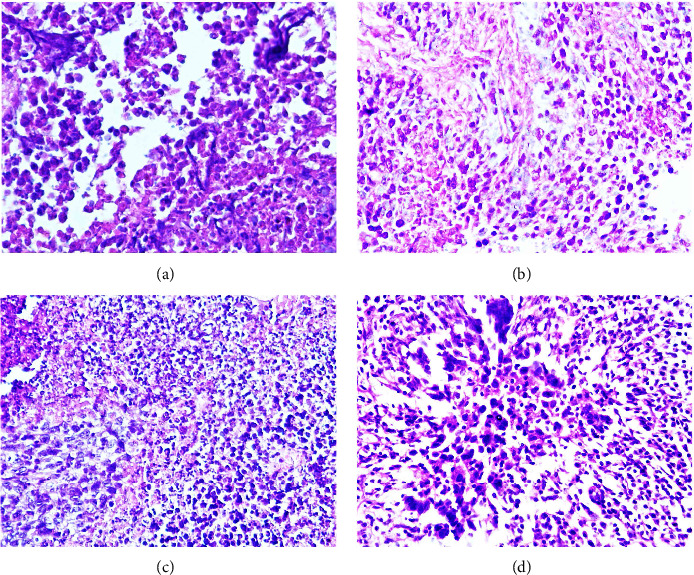
Histopathological sections analysis to the treated and untreated AN3 mouse mammary adenocarcinoma tumor (H&E). (a) Control hyperchromatic tumor cells with a high nuclear-cytoplasmic ratio (×40). (b) Tumor section in the NDV-treated group after 30 days of treatment showing tumor necrosis with tissue debris, replacement of tumor cell with fibrous connective tissue, hemorrhage, and infiltration of mononuclear cells, lymphocytes (×40). (c) Tumor section in the papaverine-treated group after 30 days of treatment shows necrotic and apoptotic tumor cells aggregation, hemorrhage and lymphocyte infiltration. Tumor section treated after the combination-treated group after 30 days of treatment with extensive necrotic area with nuclear debris and residue of the cytoplasm and a mixture of apoptotic and necrotic cells with infiltration of inflammatory cells and fibrosis. (d) Combination.

Histological examination of the sections prepared from tumor masses for the control and treated groups was investigated to analyze the antitumor effects after treatment. The histological examination of the untreated control sections showed active polyhyperchromatic tumor cells with a high nuclear-cytoplasmic ratio with the presence of multiple nuclei and mild inflammatory cells infiltration (Figure 7(a)). The tumor section of the NDV treated group showed tumor necrosis (mixture of necrotic and apoptotic cells), tissue debris, hemorrhage, and infiltration of mononuclear cells (Figure 7(b)). The papaverine-treated group's tumor section showed necrotic tumor cells aggregation and hemorrhage with lymphocyte infiltration (Figure 7(c)). Finally, for the combination (NDV-papaverine) treated group, the tissue sections revealed that there was an extensive necrotic area with nuclear debris and residue of the cytoplasm and a mixture of apoptotic and necrotic cells with infiltration of inflammatory cells and fibrosis (Figure 7(d)).

### 3.6. Quantitative Analysis of Apoptosis Proteins

**Figure 8:**
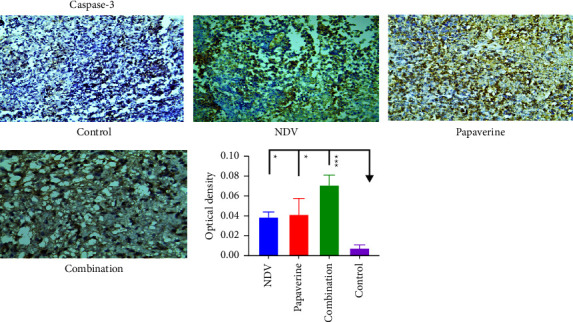
Quantification of the apoptosis proteins in tumor tissue section via immunohistochemistry assay after treatment with NDV, papaverine alone, and a combination showed a significant difference in expression of caspase-3 between NDV, papaverine, their combination, and the control untreated group with *p* value = (^*∗*^) 0.0218, (^*∗*^) 0.0141, and (^*∗∗∗*^) 0.0002, respectively.

**Figure 9:**
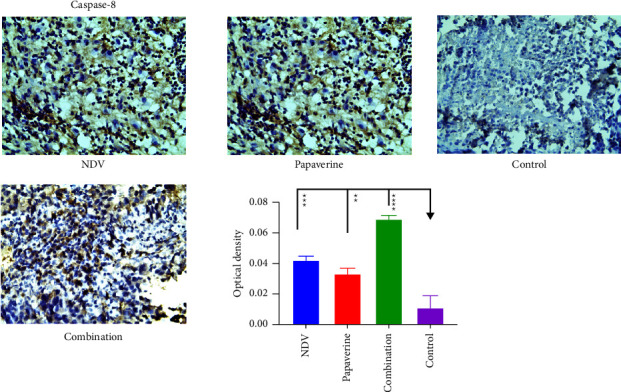
Quantification of the apoptosis proteins in tumor tissue section via immunohistochemistry assay after treatment with NDV, papaverine alone, and a combination. There was a significant difference in expression of caspase-8 between NDV, papaverine, their combination, and the control untreated group with a *p* value = (^*∗∗∗*^) = 0.0001, (^*∗∗*^), and (^*∗∗∗∗*^) = <0.0001, respectively.

**Figure 10:**
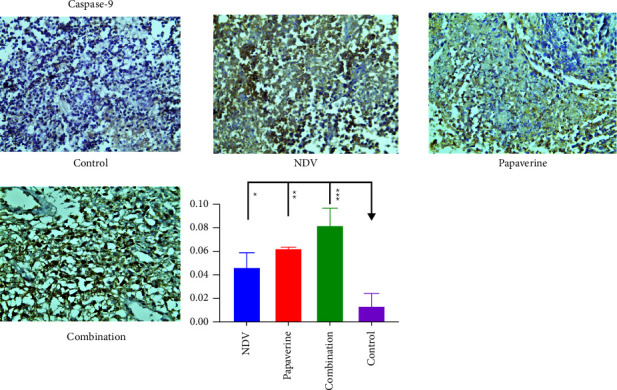
Quantification of the apoptosis proteins in tumor tissue sections via immunohistochemistry assay after treatment with NDV, papaverine alone, and a combination. There was a significant difference in expression of caspase-9 between NDV, papaverine, their combination, and the control untreated group with *p* value = (^*∗*^) = 0.0306, (^*∗∗*^) = 0.0032, and (^*∗∗∗*^) = 0.0003, respectively.

Caspase-3, caspase-8, and caspase-9 expression were evaluated in tumor specimens by immunohistochemistry assays to assess apoptosis in treated and control groups. Immunohistochemistry analysis demonstrated a significant difference in expression of caspase-3 between NDV, papaverine, their combination, and the control untreated group with a *p* value = (^*∗*^) 0.0218, (^*∗*^) 0.0141and (^*∗∗∗*^) 0.0002, respectively (Figure 8). Furthermore, a significant difference was found in caspase 8 expression between NDV, papaverine, their combination and the control untreated group with *p* value = (^*∗∗∗*^) = 0.0001, (^*∗∗*^) and (^*∗∗∗∗*^)=<0.0001, respectively (Figure 9). Also, there was a significant difference in expression of caspase 9 between NDV, papaverine, and their combination and control untreated group with *p* value = (^*∗*^) = 0.0306, (^*∗∗*^) = 0.0032, and (^*∗∗∗*^) = 0.0003, respectively (Figure 10).

### 3.7. Microarray Analysis

**Figure 11:**
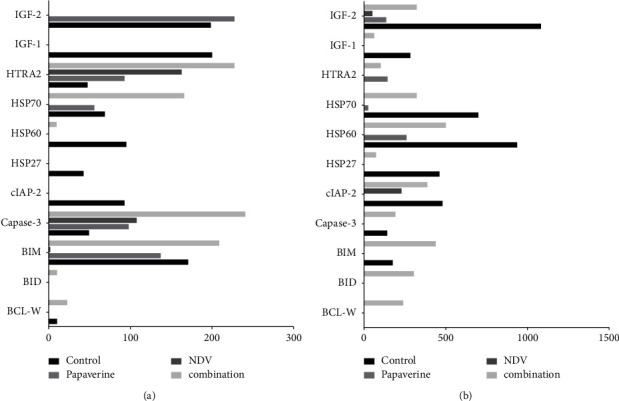
Combined therapy of papaverine-NDV activated apoptosis pathway. (a) In vitro results, using AMJ13 human breast cancer cell line, and (b) in vivo results using AN3 mouse mammary adenocarcinoma model. Microarray analysis showed an increased expression of proapoptotic proteins; BCL-W, caspase-3, BID, HTRA2, and decreased expression of antiapoptotic factors, cIAP2, IGF-1, and IGF-2, as a mechanism for the combination therapy to induce cell death.

The mechanism of combination therapy to induce cancer cell death was investigated through an apoptosis protein array that was utilized to study apoptosis protein expression in treated and untreated breast cancer cell line AMJ13 and AN3 mammary adenocarcinoma mouse models (Figures 11(a) and 11(b)). The results showed an increase in the expression of the apoptotic proteins for both in vitro and in vivo experiments, indicating that apoptosis is the main mechanism to induce cell death in treated cancer cells.

As shown in Figure 11, combined therapy of papaverine-NDV showed an increased expression of proapoptotic proteins, BCL-W, caspase-3, BID, and HTRA2, and decreased antiapoptotic factors, cIAP2, IGF-1, and IGF-2, as a mechanism for the combination therapy to induce cell death in vitro and in vivo.

## 4. Discussion

In the current study, we combined NDV with papaverine as a novel combination and compared its effect to monotherapies on *in vitro* human breast cancer cell lines and an *in vivo* mouse model to determine the effectiveness of this novel combination therapy. Naturally occurring oncolytic viruses emerge as novel tools for selective growth in and killing various tumor cells [40]. Furthermore, one of the major antitumor activities is the induction of apoptosis [41, 42]. For fully effective tumor eradication and the best chance for complete tumor destruction, it is necessary to combine its mechanism of action with other drugs with selective antitumor activity, like papaverine (nonaddictive opium derivative) with the ability of apoptosis induction [43]. Papaverine was demonstrated to inhibit human prostate cancer cell (PC-3) growth by inducing mitochondrial-mediated apoptosis, cell cycle arrest, and downregulation of the NF-*κ*B/PI3K/Akt signaling pathway [44], and vasodilating effect to increase delivery of NDV to the tumor site. Several studies have approved the antitumor effect of papaverine on various cancer cell lines [43, 45, 46]. Nevertheless, this study is the first to demonstrate the presence of synergism between papaverine and oncolytic NDV. This study aimed to evaluate the increase in the sensitivity of cancer cells to oncolytic virotherapy using papaverine and the synergistic combinations of different concentrations that enhanced the cytotoxic effect.

In vitro results showed synergistic combinations at different concentrations that enhanced the cytotoxic effect on breast cancer cell lines but not on normal cell line. The combination of papaverine and NDV had the strongest killing effect on cancer cell lines compared to monotherapies. The combination showed a superior cytotoxic effect on the breast cancer cell line with a neglecting killing effect on the normal cells. Moreover, CI values documented high synergism between papaverine and NDV (CI < 1) in MCF-7 and AMJ13 cell lines. Most values for normal cells showed no synergism with negligible cytotoxic effects due to the absence of any death percentage above 50%.

The AO/PI apoptosis assay showed that papaverine and NDV alone could induce apoptosis in cancer cells with insignificant induction in normal cells. Several studies showed that NDV and papaverine alone could induce apoptosis in treated cells [47, 48].

We conducted an in vivo study on immunocompetent mice transplanted with mouse mammary adenocarcinoma cells to investigate the in vitro results. The in vivo study confirmed that the anticancer activity of combinations was higher than that of monotherapies. The reduction in tumor volume in the combination group is more than in the NDV and papaverine groups which confirmed the in vitro results. Our current study is the first to demonstrate the presence of synergism between NDV and papaverine as a combination in vivo. To explain this synergistic enhancement of the antitumor action, we need to address that both NDV and papaverine alone can induce apoptosis, so a quantitative immunohistochemistry assay was used to study the caspase's expression levels in infected tumor tissue. The results showed that NDV had a powerful effect on inducing apoptosis in AN3 mammary adenocarcinoma with significant expression of caspase3, 8, and 9 in NDV-treated group with a *P* value (^*∗*^) 0.0218, (^*∗∗∗*^) 0.0001, and (^*∗*^) 0.0306, respectively, in comparison to the control untreated group. It was described previously [49] that NDV-mediated apoptosis involves the activation of caspase-9, followed by the activation of caspase-3 and, ultimately, the activation of caspase-8. Once activated, caspase-8 may participate in an amplification mechanism, further activating effector caspases to accelerate NDV-mediated cell death [13, 50, 51]. Caspase-8 was identified as an initiator caspase triggered by death receptor activation. Thus, the caspase-8 activation suggests that NDV can induce apoptosis through the extrinsic death receptor pathway.

Furthermore, infection led to the loss of mitochondrial membrane and activation of caspase-9, highlighting the intrinsic pathway's importance in activating NDV-mediated apoptosis [52]. Activation of caspase-3 induces the cleavage of protein kinases, cytoskeletal proteins, DNA repair proteins, and finally, destruction of “housekeeping” cellular functions. Caspases also affect the cytoskeletal structure, cell cycle regulation, and signaling pathways, ultimately leading to the morphologic manifestations of apoptosis, such as DNA condensation and membrane blebbing [53]. Moreover, papaverine showed efficient apoptosis induction in the papaverine-treated group, in which there was a significant level of caspase 3, 8, and 9 with *p* value (^*∗*^) 0.0141, (^*∗*^) 0.0141, and (^*∗∗*^) 0.0032 in comparison to the control untreated group. That agreed with multiple previous studies that approved the ability of papaverine to induce apoptosis [43, 45, 48].

While for the combination group, quantitative immunohistochemistry assay results revealed synergism in apoptosis induction with higher caspases level in combination than the untreated control group with *P* value (^*∗∗∗*^) 0.0002, (^*∗∗∗∗*^) <0.0001, (^*∗∗∗*^) 0.0003 for caspase-3, 8, and 9 in which the expression of these caspases was significantly higher than monotherapies.

Microarray analysis findings support in vitro and in vivo results in which NDV, papaverine, and combinations can induce apoptotic factors within the first 24 h after exposure. This effect was higher in combination-treated groups, which may explain a higher cytotoxic effect on breast cancer cell lines in vitro and a higher growth inhibition effect on tumor-bearing mice in vivo. Also, microarray results show the ability of the papaverine-NDV combination to induce increased.

To investigate the molecular mechanisms of apoptosis in response to NDV infection, papaverine, and their combination, the apoptosis proteins array was conducted in treated and untreated AMJ-13 and on tumor tissue from AM3 tumor-bearing mice.

We found that combined therapy of the papaverine-NDV induced an increased expression of proapoptotic proteins (BCL-W, caspase-3, BID, and HTRA2) and decreased expression of antiapoptotic factors (cIAP2, IGF-1, IGF-2, and HSPs) as a mechanism for the combination therapy to induce cell death in vitro and in vivo.

That can explain significant apoptotic death cells in treated groups due to caspase elevation and elevation of other essential apoptotic proteins.

Antiapoptotic proteins (apoptosis gatekeepers, such as BCL-2 and BCL-w): several clinical correlative studies explained the elevated level of antiapoptotic Bcl-2 proteins, and survivin is responsible for the resistance of breast cancer cells [54]. Various evidence has suggested that most chemotherapeutic agents exert their anticancer activity by inducing apoptosis; therefore, antiapoptosis may be a major factor limiting their effectiveness [55]. Interestingly, it was found that the same stress signals that trigger apoptosis and increase apoptotic proteins also stimulate the expression and release of heat shock proteins (HSPs) [56]. HSPs are molecular chaperones (protective proteins) normally over-expressed by cells when exposed to protein denaturation, such as extreme temperatures, hypoxia, heavy metals, drugs such as papaverine, and viral infections NDV or their combination [57]. That demonstrates the elevation of an essential apoptotic protein and HSPs in our study. However, numerous mechanisms explain the cytoprotective effects of HSPs against apoptosis, one of them being the ability of HSPs to interact with cytochrome c and block its dimerization with Apaf-1, hence preventing the formation of the apoptosome complex or through inhibition of proapoptosis factors, such as p53, Bax, and Bid [44]. Thus, downregulating HSPs can have therapeutic value to help induce apoptosis [58].

Another remarkable finding is that the reduction in expression of insulin-like growth factor-1 (IGF-1 and 2) in vitro and in vivo has very important therapeutic value, as it has been reported that the IGF-1-stimulated signal transduction pathway stimulates breast cancer cell proliferation, survival, and metastasis. [59]. Our results reported downregulation of cIAP2 after papaverine-NDV combined therapy in vitro and in vivo, which effectively boosts apoptosis by triggering caspase 3/7 in cancer cells. This makes cIAP2 inhibition of therapeutic value [60]. Caspase-3's increased expression confirms the ICC finding of our experiments in vitro and in vivo.

In conclusion, NDV is an attractive anticancer agent when combined with other antitumor agents. It was found to be synergistic with papaverine at low doses on breast cancer cell lines in vitro and AN3 mammary adenocarcinoma in vivo through caspase induction involvement and their ability to induce a wide range of apoptotic proteins. These results indicate a novel combination that can enhance the antitumor ability of both oncolytic virotherapy and papaverine.

## Figures and Tables

**Table 1:** The cotoxic effect and synergism effect of NDV and papaverine as a combination treatment on mcf-7 cells.

Dose NDV	Dose pap	Effect	Combination index CI	Effect
0.1	10.0	0.778	0.16769	Synergism
0.1	25.0	0.829	0.18264	Synergism
0.1	50.0	0.884	0.11521	Synergism
1.0	10.0	0.846	0.05338	Synergism
1.0	25.0	0.854	0.11329	Synergism
1.0	50.0	0.863	0.18736	Synergism
2.0	10.0	0.849	0.05052	Synergism
2.0	25.0	0.857	0.10662	Synergism
2.0	50.0	0.877	0.13661	Synergism

**Table 2:** The cotoxic effect and synergism effect of NDV and papaverine as a combination treatment on AMJ-13 cells.

Dose NDV	Dose papaverine	Effect	Combination index CI	Effect
0.1	10.0	0.609	0.82597	Synergism
0.1	25.0	0.654	1.00007	Additive
0.1	50.0	0.699	0.92518	Synergism
1.0	10.0	0.632	0.57297	Synergism
1.0	25.0	0.685	0.59162	Synergism
1.0	50.0	0.754	0.32724	Synergism
2.0	10.0	0.667	0.32197	Synergism
2.0	25.0	0.728	0.27184	Synergism
2.0	50.0	0.836	0.04870	Synergism

**Table 3:** The cotoxic and synergism effects of NDV and papaverine as a combination treatment on normal cells.

Dose NDV	Dose pap	Effect	Combination index CI	Effect
0.1	10.0	0.182	0.35777	Synergism
0.1	25.0	0.208	0.84347	Synergism
0.1	50.0	0.244	1.80122	Antagonism
1.0	10.0	0.203	1.23505	Antagonism
1.0	25.0	0.214	1.74186	Antagonism
1.0	50.0	0.226	2.61146	Antagonism
2.0	10.0	0.18	1.99904	Antagonism
2.0	25.0	0.26	3.15104	Antagonism
2.0	50.0	0.26	4.04236	Antagonism

## Data Availability

The data used to support the findings of this study are available from the corresponding author upon request.
